# Brain tumor segmentation using a combination of graph cut and optimized U-Net with genetic algorithm: An efficient hybrid framework for enhanced medical image analysis

**DOI:** 10.1371/journal.pone.0338773

**Published:** 2026-10-14

**Authors:** Reem M. Mostafa, Emad Mabrouk, Mohamed Saed Salim, Ahmed Ayman, Hamdy Z. Zidan

**Affiliations:** 1 Department of Mathematics, Faculty of Science Al-Azhar University, Assiut, Asyut, Egypt; 2 College of Engineering and Technology, American University of the Middle East, Egaila, Kuwait; Università degli Studi di Napoli Federico II: Universita degli Studi di Napoli Federico II, ITALY

## Abstract

Accurate segmentation of low-grade gliomas (LGGs) in FLAIR MRI remains challenging due to low-contrast tumor boundaries, irregular morphology, and extreme class imbalance. This work presents a lightweight hybrid segmentation framework that integrates three complementary components: (1) a fast graph-based region refinement step using GrabCut applied during training only to enhance tumor–background contrast, (2) a compact U-Net architecture whose filter configuration is automatically selected using a small-scale genetic algorithm, and (3) an analytical evaluation of lightweight post-processing strategies studied separately from the final GA-optimized model. The method was evaluated on the LGG-MRI-Segmentation dataset using strict patient-level splits of 79 training, 19 validation, and 12 testing patients. The final model achieved strong slice-wise performance with a mean Dice of 0.9132 and a median Dice of 0.9855; the lower mean reflects the influence of more difficult, lower-performing slices and therefore provides a more conservative summary of overall performance across the evaluated test cases. Additional metrics include an IoU of 0.8851, sensitivity of 0.8954, precision of 0.9523, specificity of 0.9997, and an HD95 of 1.00 mm, indicating highly accurate boundary delineation. For contextual benchmarking, a 2D nnU-Net model was trained on the same dataset. nnU-Net achieved a mean validation Dice of 0.8450 and a best validation EMA Dice of 0.9134. Because these nnU-Net Dice values were obtained from its validation workflow rather than the exact slice-wise test procedure used for the proposed model, they are presented as contextual reference results rather than strictly matched test-set estimates. The proposed framework required substantially less training time under the evaluated implementations. Taken together, these findings demonstrate that a carefully coordinated combination of preprocessing, lightweight architectural optimization, and efficient post-processing can achieve strong segmentation performance on the evaluated dataset while remaining computationally efficient. These findings suggest that the proposed framework is promising for computationally constrained LGG segmentation workflows. However, broader clinical applicability requires validation on independent external cohorts before generalizability can be established.

## Introduction

Brain tumors are broadly classified into several types based on their cellular origin, with gliomas (arising from glial cells), meningiomas (arising from the meninges), and pituitary tumors being the most common primary brain neoplasms. Among these, gliomas–-particularly low-grade gliomas (LGG)–-present unique clinical challenges due to their infiltrative growth patterns, tendency to progress to higher grades, and critical anatomical locations that complicate surgical resection. These tumors exhibit heterogeneous appearance, poorly defined margins on conventional imaging, and variable prognosis depending on histological grade and molecular markers. Precise tumor segmentation from MRI scans is essential for multiple clinical applications: treatment planning, radiotherapy targeting, surgical navigation, and tracking disease progression over time. Currently, radiologists manually outline tumors–-a process that’s not only time-consuming but also subjective and difficult to standardize across different readers. This variability has driven strong interest in developing automated segmentation systems that are both reliable and practically applicable [1,2].

For low-grade gliomas (LGG), Fluid-Attenuated Inversion Recovery (FLAIR) MRI sequences are particularly valuable because they highlight these lesions as hyper-intense regions. However, creating automated tools to segment LGG in FLAIR images remains surprisingly difficult. The obstacles are several: tumor boundaries typically show minimal contrast against surrounding healthy tissue, tumor morphology varies dramatically between patients, non-tumor background pixels vastly outnumber tumor pixels creating extreme class imbalance, and infiltrative tumor margins often blend subtly with normal brain parenchyma [3,4].

The rise of deep learning has brought significant advances, with U-Net [5] and its numerous descendants [6–9] establishing themselves as the foundation for most modern segmentation approaches. However, for segmenting LGG in FLAIR MRI specifically, progress is slow because of three key challenges.

The first issue concerns insufficient preprocessing. Conventional normalization approaches like z-score standardization or histogram equalization were designed for general image enhancement tasks. When applied to FLAIR images of LGG, they often fail to sufficiently enhance the subtle intensity differences that distinguish tumor boundaries, particularly for small or diffusely infiltrating lesions [10]. Essentially, the segmentation model receives input data where the most critical features–-the tumor edges–-remain poorly defined from the start.

A second challenge involves the non-systematic way most U-Net architectures are designed. Critical parameters including network depth, filter progression patterns, and activation function choices are typically selected based on conventions borrowed from other computer vision domains or through limited empirical testing [11]. This approach means the massive architectural search space is rarely explored systematically, likely resulting in models that are either unnecessarily complex or inadequately powerful for the specific demands of LGG segmentation.

Third, the computational burden of latest methods creates a major barrier to clinical adoption. Frameworks like nnU-Net [12] deliver impressive benchmark performance but come with substantial costs: extensive GPU time requirements for multi-stage training, significant memory demands that exceed what’s available in many clinical settings, and complex configuration procedures. These factors together limit their practical use in real-world clinical environments where solutions need to be rapid, low-cost, and straightforward to deploy.

Compounding these issues, many top-performing methods assume availability of multiparametric MRI data (T1, T1ce, T2, and FLAIR) as demonstrated in BraTS benchmark competitions [13,14]. In actual clinical practice, however, LGG assessment often relies on FLAIR sequences alone. Meanwhile, newer transformer-based architectures [15,16] show exciting potential but come with even greater computational demands that further restrict their practical deployability.

**The proposed contribution** tackles these challenges head-on, by developing a lightweight hybrid framework specifically designed for LGG segmentation in FLAIR MRI. The key elements of the proposed approach include:

1. **Graph-cut refinement:** by employing a fast GrabCut-based preprocessing step that sharpens tumor-background separation, but crucially applying it only during training to avoid any inference-time computational overhead [17,18].
2. **Architecture optimization:** by creating a compact U-Net variant where the filter configuration is automatically determined using a computationally efficient, small-scale genetic algorithm (GA) [19,20].
3. **Performance demonstration:** by reporting the segmentation performance and computational characteristics of the proposed streamlined pipeline on the evaluated dataset, with nnU-Net included as a contextual reference experiment.

Beyond these individual components, the core idea of this work is to develop a practical and computationally lightweight segmentation pipeline tailored specifically to FLAIR-only LGG imaging–-a setting commonly encountered in real clinical workflows. This focus is important because many hospitals do not routinely acquire full multiparametric MRI sequences, and heavy frameworks such as nnU-Net remain difficult to train, deploy, and maintain in resource-constrained environments. By designing a fast, computationally efficient model intended for practical deployment, the proposed method addresses a critical gap between academic research systems and practically feasible solutions.

The proposed method was evaluated using rigorous patient-level splits on a public LGG dataset. A 2D nnU-Net experiment was included as a contextual reference; however, because its reported Dice values were obtained from its validation workflow rather than the exact slice-wise test procedure used for the proposed model, these values are not interpreted as a strictly matched head-to-head benchmark. The manuscript also contextualizes the proposed method against representative classical, CNN-based, transformer-based, hybrid, and lightweight segmentation approaches reported in the literature.

## Related work

### CNN-based segmentation methods

Convolutional neural networks have become the dominant technological approach for brain tumor segmentation, with the U-Net architecture [5] serving as the foundation for most contemporary methods. Numerous research groups have built upon this core design. Iqbal and colleagues [21] developed a multiparametric CNN that achieved Dice scores around 0.86 on the BraTS 2015 dataset. Zhao et al. [13] demonstrated that combining predictions from multiple 3D U-Nets through ensemble methods improved segmentation consistency in multiparametric BraTS tasks. Meanwhile, Chen et al. [22] applied 3D convolutional networks to delineate multiple glioma subregions simultaneously, reporting Dice scores as high as 0.95 for enhancing tumor components. Mahyoub et al. [23] introduced a residual CNN tailored for FLAIR-only LGG segmentation, reporting a Dice score of 0.843, though using a non-patient-level split strategy.

Ensemble-based segmentation strategies have also demonstrated strong performance in brain tumor analysis by combining predictions from multiple complementary models. Such approaches often improve consistency and reduce prediction variance, particularly in heterogeneous tumor regions. For example, ensemble learning has been explored in large-scale BraTS benchmark studies where multiple U-Net variants or multi-view architectures are fused to improve segmentation stability and boundary consistency. While these methods can achieve excellent segmentation accuracy, they typically introduce substantially higher computational and memory requirements during both training and inference, which may limit their practical deployment in resource-constrained clinical environments.

More recent architectural innovations have focused on capturing multi-scale contextual information. The DMFNet approach [14] incorporated dilated convolutions to maintain computational efficiency while expanding receptive fields. UNet++ [6] introduced nested skip connections to refine feature propagation between encoder and decoder pathways. Most recently, transformer-based models like Swin-UNet [15] and UTNet [16], along with various hybrid CNN-transformer designs, have pushed performance further by improving global context modeling. At the same time, there has been increasing interest in computationally efficient segmentation architectures that attempt to balance accuracy with deployability. Lightweight encoder-decoder networks, compact residual models, and efficiency-oriented hybrid systems aim to reduce parameter count, memory usage, and training time while preserving clinically acceptable segmentation quality. This trend is particularly important for medical institutions with limited GPU infrastructure or workflows relying on rapid inference and simplified deployment. Despite their impressive results, these advanced models typically share several requirements that limit their clinical utility for LGG: they depend on multiparametric MRI input, need extensive training data, and demand substantial GPU resources–-conditions that frequently don’t align with real-world LGG clinical workflows where FLAIR-only scans are common and computational resources may be constrained.

Complementary to automated segmentation methods, standardized neuroimaging descriptor frameworks can support reproducible characterization of glioma imaging phenotypes. Negro et al. introduced the updated VASARI 2.0 MRI lexicon for standardized assessment of imaging features associated with glioma grade and IDH status [24].

### Graph-based segmentation approaches

Graph-based methodologies have a long history in medical image segmentation due to their ability to enforce spatial consistency and incorporate smoothness priors directly into the segmentation process. The GrabCut algorithm and related graph-cut formulations have been successfully applied to MRI segmentation tasks, typically by modeling tumor and background intensity distributions using Gaussian mixture models [17]. In a recent example, Soloh et al. [18] proposed an α-expansion graph cut technique specifically for brain tumor segmentation, reporting high accuracy for both low-grade and high-grade glioma cases.

Optimization-driven region-growing and graph-based segmentation strategies have also been investigated to improve boundary adaptation and segmentation consistency in weak-boundary medical imaging scenarios [25,26]. In addition, evolutionary optimization techniques have previously been explored for improving graph-based medical image segmentation under challenging boundary conditions. Mostafa et al. [27] proposed a genetic programming framework for evolving adaptive unary costs in graph-cut segmentation, demonstrating improved consistency and boundary delineation performance.

While graph-based methods excel at producing clean, spatially coherent boundaries, they suffer from two significant limitations: their performance depends heavily on proper initialization, and they lack the ability to learn deep contextual or textural features from data. These limitations have motivated the development of hybrid frameworks that leverage graph-based techniques as a preprocessing step to enhance the training of CNN models, while avoiding graph-based computation during actual inference when speed is critical.

### Hybrid and optimization-driven models

The field has seen growing interest in approaches that combine CNNs with complementary techniques from classical image processing, clustering algorithms, or auxiliary machine learning models. Dogra et al. [17] integrated fuzzy graph-cut refinement techniques to improve brain tumor segmentation performance. Yang et al. [28] evaluated support vector machine (SVM) and convolutional neural network (CNN) approaches for LGG MRI segmentation, highlighting the trade-off between computational efficiency and segmentation accuracy across classical and deep learning paradigms. More recently, Behzadpour et al. [29] introduced a ResUNet architecture enhanced with channel attention mechanisms, demonstrating improved boundary precision on the raw TCGA-LGG dataset, which differs from the curated LGG-MRI-Segmentation dataset used in this study.

Another interesting direction involves integrating optimization algorithms directly with deep network training and medical image segmentation workflows. Evolutionary and immune-inspired optimization techniques have previously demonstrated promising results for adaptive medical image segmentation and intelligent boundary refinement under challenging imaging conditions [26]. Genetic algorithms specifically have been employed for hyperparameter tuning and model selection in medical imaging applications [30]. However, full-scale neural architecture search (NAS) remains computationally prohibitive for most research groups, making lightweight GA-based tuning an attractive middle ground for resource-constrained environments.

### Automated configuration frameworks

The nnU-Net framework [12] has emerged as arguably the most influential automated configuration system in medical image segmentation. Its strength lies in dynamically adapting preprocessing strategies, architectural parameters, and training procedures to specific dataset characteristics. Despite its powerful performance, nnU-Net comes with significant practical drawbacks: training typically requires up to 1000 epochs, involves heavy GPU utilization, and demands large memory footprints–-factors that render it less suitable for clinical environments with limited computational infrastructure.

The principles of automated machine learning (AutoML) have been increasingly recognized for streamlining data-driven application development across various domains. As highlighted by Addula [31], AutoML frameworks automate critical tasks such as data preparation, feature selection, model selection, and hyperparameter tuning–-reducing the human expertise required while accelerating development cycles. This aligns with the growing interest in making machine learning more accessible and efficient for specialized applications.

Neural architecture search methodologies more broadly–-including reinforcement learning, evolutionary algorithms, and gradient-based optimization techniques–-have been investigated extensively [11]. Recent studies have additionally explored ensemble learning strategies, lightweight segmentation frameworks, and computationally efficient hybrid architectures to improve the balance between segmentation performance and deployment practicality. However, most comprehensive NAS frameworks require thousands of GPU hours to complete, effectively limiting their practicality to well-funded research institutions with substantial computing resources.

### Positioning of the proposed method

The existing research landscape clearly demonstrates that classical graph-based approaches, CNN architectures, transformer-based systems, automated configuration frameworks, and hybrid optimization-driven models can all generate high-quality tumor segmentations under appropriate conditions. However, these methods differ substantially in computational complexity, training cost, data requirements, interpretability, and deployment practicality. Several critical gaps nevertheless remain that significantly hinder clinical adoption.

Compared with classical segmentation approaches, the proposed framework benefits from the feature-learning capability of CNNs while preserving lightweight computational characteristics through compact architectural design. Compared with transformer-based and large automated frameworks, the proposed method prioritizes reduced computational burden, faster training, and simplified deployment. Unlike many recent studies that rely on multiparametric MRI input or extensive architecture search procedures, the present work focuses specifically on practical FLAIR-only LGG segmentation under constrained computational settings. This positioning is important because benchmark-leading performance alone does not necessarily translate into practically applicable clinical systems, particularly in environments with limited GPU resources or incomplete MRI acquisition protocols. Unlike many recent studies that primarily prioritize maximum benchmark accuracy through increasingly large architectures, ensemble systems, or computationally intensive optimization pipelines, the proposed work emphasizes practical efficiency and deployment simplicity. The novelty of the present framework does not arise from introducing a radically new deep architecture, but rather from the coordinated integration of three lightweight components: training-only graph-cut refinement, compact GA-guided architectural optimization, and efficient FLAIR-only segmentation. This combination specifically targets the often-overlooked balance between segmentation quality, computational cost, and real-world deployability.

Perhaps the most fundamental limitation is that the majority of high-performing models depend on multiparametric MRI data, typically leveraging the complementary information available from T1, T1-contrast enhanced (T1ce), T2, and FLAIR sequences as standardized in high-profile datasets like the Brain Tumor Segmentation (BraTS) challenge. While this multiparametric approach certainly provides richer information that boosts segmentation accuracy by capturing different tissue properties, its clinical utility is fundamentally limited because many routine LGG clinical scans provide only FLAIR sequences. This sequence has become a workhorse in clinical neuro-oncology due to its particular sensitivity for LGG detection, but building methods that rely on it alone presents challenges that many widely-recognized models aren’t designed to handle. Consequently, there’s a pressing need for segmentation approaches that can deliver robust, reliable performance using only the widely available FLAIR sequence, which would dramatically improve their potential applicability in future studies.

Another concerning trend has been the development of increasingly heavy architectures, including complex 3D CNNs and transformer models with elaborate attention modules. While these designs are definitely powerful, they require considerable GPU resources to train and deploy. These models often contain tens or even hundreds of millions of parameters, demanding high-end GPUs with substantial memory capacity for both training and inference. This computational burden makes them inaccessible to many research laboratories and, more importantly, to clinical institutions with limited computational budgets, thereby hindering the democratization of these advanced tools.

Similarly, fully automated frameworks like nnU-Net provide impressive accuracy but demand long training times and high memory capacity. The extensive training regimens–-which can span several days–-coupled with the large model footprints of these systems create a substantial barrier to entry for rapid prototyping and deployment in resource-constrained clinical settings.

Finally, despite its proven potential for creating efficient, specialized models in other domains, lightweight genetic algorithm-based architecture tuning remains surprisingly underexplored for brain tumor segmentation. While genetic algorithms have seen successful application in broader neural architecture search contexts, their specific use for creating computationally inexpensive yet powerful U-Net variants in medical imaging hasn’t been thoroughly investigated. This represents a significant missed optimization opportunity, particularly for developing models that could be tailored to specific hardware constraints or latency requirements without sacrificing segmentation performance.

Motivated directly by these identified gaps, the present study introduces a computationally efficient hybrid pipeline that combines graph-cut preprocessing, a compact U-Net refined through lightweight genetic algorithm search, and straightforward morphological post-processing. By combining these elements, the method demonstrates strong internal segmentation performance and reduced computational requirements on the evaluated dataset, while the nnU-Net experiment provides contextual reference information.

## Materials and methods

### Dataset description

All experiments were conducted on the LGG-MRI-Segmentation dataset using a single NVIDIA A100 GPU (40GB memory) through Google Colab Pro + , sourced from The Cancer Imaging Archive (TCIA) [10,32]. This collection includes 3,929 axial FLAIR MRI slices obtained from 110 patients diagnosed with WHO grade II–III low-grade gliomas. Every MRI slice comes with a corresponding manually annotated binary tumor mask that serves as ground truth for training and evaluation.

Strict patient-level splitting has been implemented to prevent any potential data leakage that could artificially inflate the performance metrics. This meant that all slices from a single patient were assigned exclusively to one of three subsets:

- Training: 79 patients (72%)
- Validation: 19 patients (17%)
- Testing: 12 patients (11%)

This careful partitioning ensures that the model’s performance on the test set genuinely reflects its ability to generalize to completely new patients rather than simply recognizing variations of images it encountered during training. A concise summary of these dataset characteristics is provided in Table 1.

**Table 1:** Key characteristics of the LGG-MRI-Segmentation dataset used in the study.

Total patients	110
Total slices	3,929
Modality	FLAIR MRI
Train patients	79 (72%)
Validation patients	19 (17%)
Test patients	12 (11%)
Resolution	256×256

Using patient-level splits is intentionally more challenging than slice-level evaluation, but it provides a more realistic assessment of performance on previously unseen patients. In practical settings, a segmentation model must generalize to entirely new patients with different anatomy, scanner characteristics, and acquisition conditions. Ensuring that no slices from the same individual appear in both training and testing eliminates hidden correlations and provides a realistic measure of true clinical performance within the evaluated dataset.

Before feeding images to the model, each MRI slice went through a standardized preprocessing pipeline designed to improve data quality and consistency:

- N4ITK bias-field correction to address intensity inhomogeneities common in MRI scans
- Skull stripping to remove non-brain tissues and focus on the relevant brain parenchyma
- Z-score normalization applied independently to each slice to standardize intensity ranges
- Resizing to a uniform 256×256 resolution to ensure consistent input dimensions
- Conversion of segmentation masks to a binary format using a fixed threshold of 0.5.

This research uses fully anonymized, publicly accessible MRI data from The Cancer Imaging Archive (TCIA). No identifiable patient information is included, and according to institutional guidelines, no additional ethical approval was required.

### Graph-cut preprocessing (training only)

To help the model better distinguish tumor regions from background tissue, a graph-cut refinement step has been incorporated exclusively during the training phase. This design choice enhances tumor visibility during learning without introducing any computational overhead during inference when speed matters most.

This implementation was performed using the GrabCut algorithm, which minimizes the standard graph-cut energy as defined in Eq. (1) [33]:


$$\begin{array}{c} E(f)=\sum_{p\in P}D_{p}(f_{p})+\lambda \sum_{(p,q)\in N}V_{p,q}(f_{p},f_{q}) \end{array}$$ (1)

Here, $f_{p}\in \{0,1\}$ represents the binary label (tumor or background) for each pixel *p*, while $D_{p}$ constitutes the data term that was modeled using Gaussian intensity distributions derived from ground truth masks. The $V_{p,q}$ term enforces spatial smoothness by encouraging neighboring pixels to share similar labels. In the experiments, the regularization weight was set λ = 25, which was found to provide a good balance between regional fidelity and boundary smoothness. Selectively rescaling image intensities further improved tumor prominence:

- Tumor core regions: multiplied by 1.15
- Tumor boundary areas: multiplied by 1.10
- Background regions: multiplied by 0.95

The preprocessing stage was completed by applying a bilateral filter to reduce noise while carefully preserving important edge information. It’s worth reiterating that this entire step has been completely omitted during testing to maintain fast inference speeds.

### U-Net architecture

The segmentation model builds upon a lightweight U-Net architecture that maintains computational efficiency while preserving strong representational capacity. The network consists of four encoder stages paired with four decoder stages, creating a symmetric structure that’s become standard for segmentation tasks. An overview of the U-Net encoder–decoder architecture with skip connections is shown in Fig 1.

**Fig 1:**
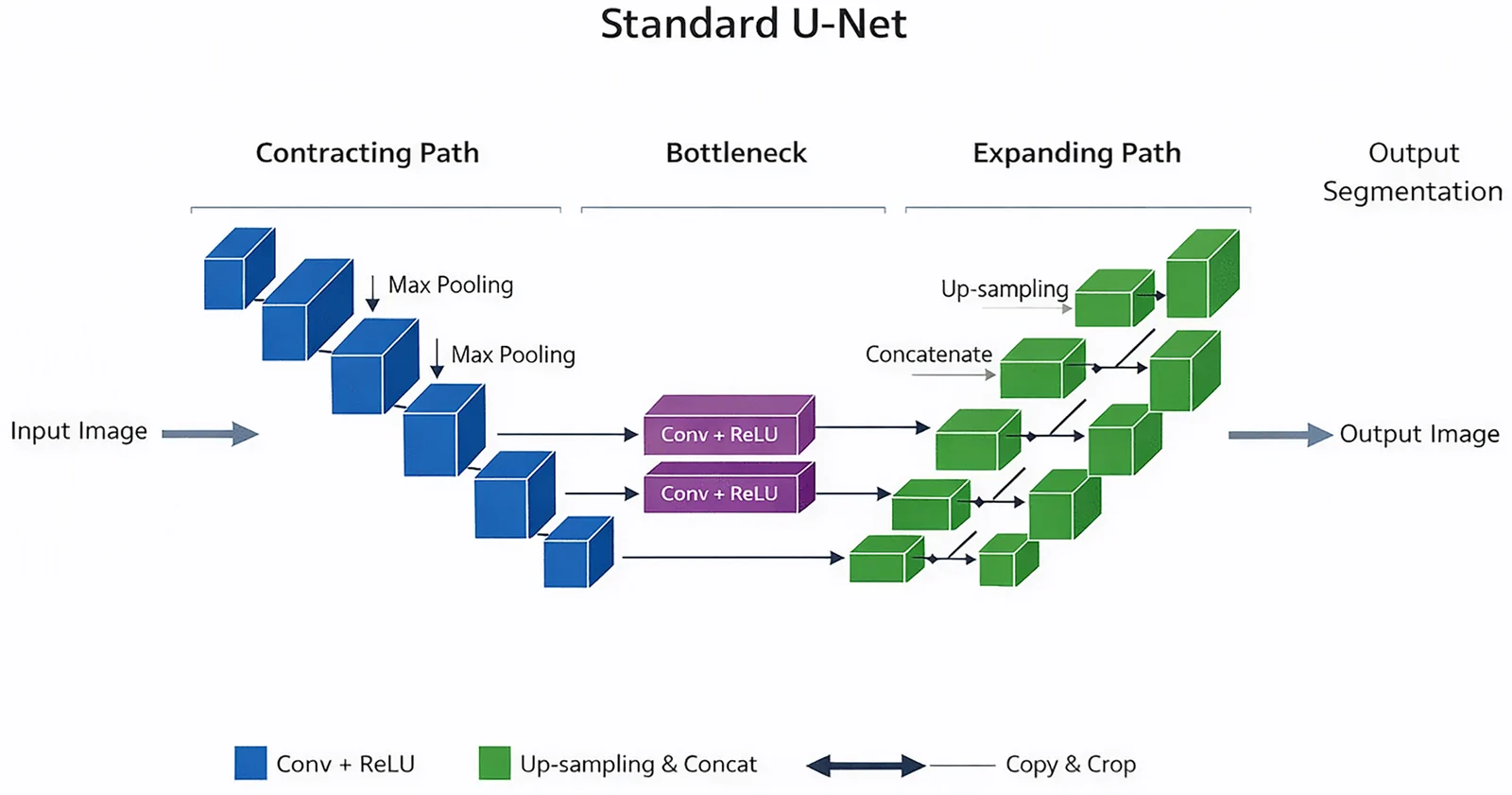
Standard U-Net architecture used as the backbone of the proposed segmentation model. The network follows an encoder–decoder structure with a contracting path for feature extraction, a bottleneck, and an expanding path with skip connections for precise spatial localization.

Each stage in the U-Net contains several key components:

- Two consecutive 3×3 convolutional layers that form the core feature extraction units
- Batch normalization layers that stabilize and accelerate the training process
- Swish or SELU activation functions in the encoder path (letting the genetic algorithm choose between these options)
- Swish or ReLU activations in the decoder path (again, selected through the optimization process)

For reducing spatial dimensions in the encoder, standard max pooling operations have been applied. In the decoder, transpose convolutions were employed to gradually upsample feature maps back to the original input resolution. The network culminates in a 1×1 convolutional layer with sigmoid activation that produces the final binary segmentation mask. The convolutional block design follows the conventional U-Net principle that pairs two sequential 3×3 convolutions with batch normalization and nonlinear activation. This structure has two advantages: (1) the stacked convolutions expand the effective receptive field while maintaining computational efficiency, and (2) batch normalization stabilizes training by reducing internal covariate shifts. Using Swish or SELU activations in the encoder encourages smoother gradient propagation, while the decoder’s ReLU/Swish activations enhance feature reconstruction during upsampling. Together, these choices create a lightweight yet expressive feature extractor well-suited for the subtle intensity variations that characterize LGG boundaries. An overview of this convolutional block design is illustrated in Fig 2.

**Fig 2:**
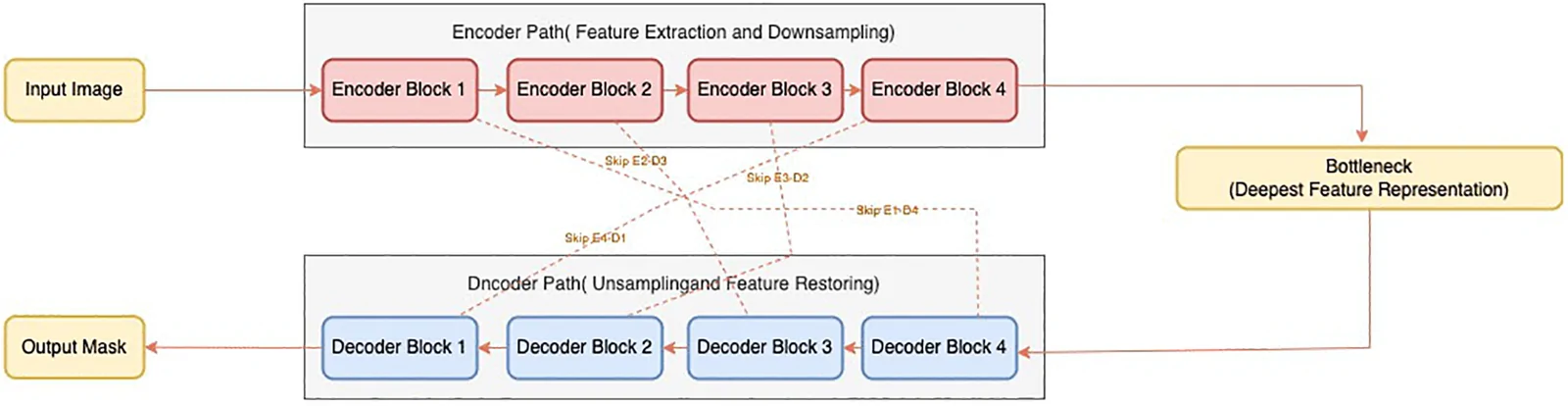
The basic convolutional block that forms the building unit throughout the U-Net architecture.

### Genetic algorithm for architecture search

Rather than relying on subjective judgment or manual hyperparameter tuning for selecting the optimal number of convolutional filters at each depth of the U-Net, a lightweight genetic algorithm was implemented to automatically explore this architectural design space in a reproducible and computationally efficient manner. The GA was deliberately designed as a small-scale evolutionary search to ensure feasibility under limited computational resources while still enabling systematic optimization of network capacity.

For chromosome encoding and architectural mapping, each candidate U-Net architecture is encoded as a fixed-length chromosome of the form:


$$\left[f_{1},f_{2},f_{3},f_{4},f_{5}\right]$$

where each gene represents the number of convolutional filters at a specific network depth. The mapping between chromosome genes and the U-Net components is explicitly defined in Table 2. The first four genes (*f*_1_ to *f*_4_) correspond to successive encoder blocks from shallow to deep features, while *f*_5_ defines the bottleneck layer that captures the most abstract representation. The decoder mirrors this encoder configuration symmetrically through skip connections.

**Table 2:** Mapping between chromosome genes and U-Net components.

Gene	U-Net Level	Functional Role
*f*_1_	Encoder Block 1	Low-level spatial features
*f*_2_	Encoder Block 2	Mid-level texture features
*f*_3_	Encoder Block 3	Deep semantic features
*f*_4_	Encoder Block 4	High-level contextual features
*f*_5_	Bottleneck Layer	Latent representation

This encoding allows the genetic algorithm to directly control the model capacity and feature hierarchy while keeping all other architectural components (kernel size, number of levels, skip connections, optimizer, and loss function) fixed for controlled experimentation. Each candidate architecture is evaluated using the Dice loss function [34]:


$$\begin{array}{c} {ℒ}_{\text{Dice}}=1-\frac{2|P\cap G|}{\left|P|+|G\right|} \end{array}$$ (2)

where *P* and *G* denote the predicted and ground truth tumor masks, respectively. Since minimizing Dice loss is equivalent to maximizing Dice overlap, this fitness directly optimizes the most clinically relevant segmentation metric.

For the evolutionary configuration, the genetic algorithm parameters were fixed as follows:

- Population size: 10 individuals
- Number of generations: 6
- Mutation rate: 0.2 (20%)
- Elite size: 2 (best individuals preserved per generation)
- Training epochs per individual: 8 epochs

Each candidate is trained for only 8 epochs using identical optimization settings to ensure fair fitness comparison while maintaining rapid evaluation. This short training schedule was sufficient to reveal relative performance differences between architectures while keeping the total search time efficient.

Moving to selection, crossover, and mutation. At each generation, individuals are ranked according to their validation Dice score. Elitism ensures that the top two individuals are carried unchanged into the next generation. The remaining offspring are generated through uniform crossover, where each gene in the offspring chromosome is sampled independently from one of the two parents with equal probability. Gaussian mutation is then applied to individual genes with probability 0.2 to introduce controlled stochastic changes and prevent premature convergence.

These evolutionary operators balance exploitation of strong filter configurations and exploration of new architectural variations. The crossover and mutation mechanisms are visualized in Fig 3.

**Fig 3:**
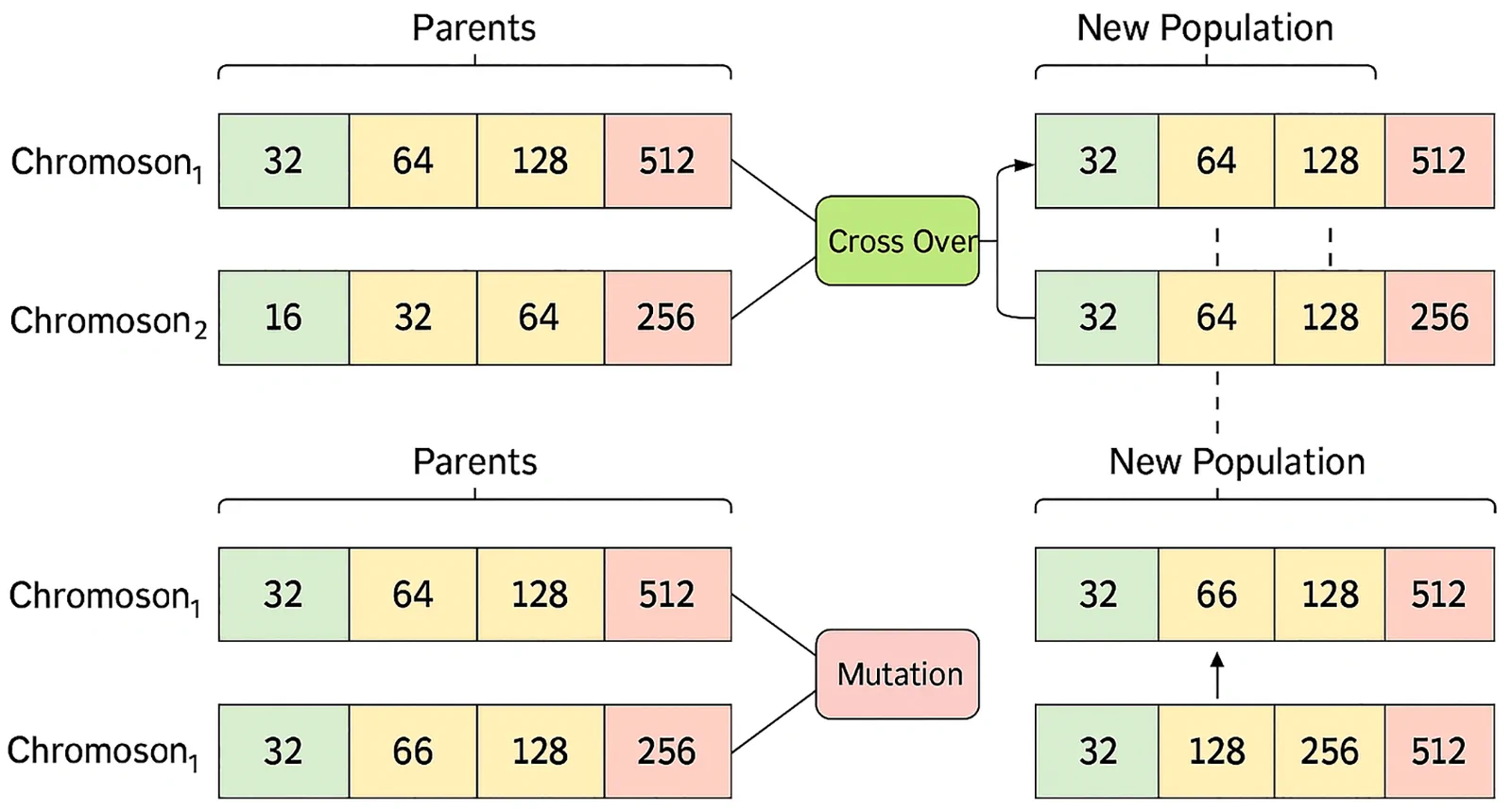
Visualization of the crossover and mutation operations used to generate new candidate architectures from existing ones.

The genetic algorithm was executed for a fixed number of generations using the configuration described above. The convergence behavior, runtime characteristics, and generation-wise fitness distributions are reported and analyzed in the Results section.

After completion of the evolutionary search, a single architecture was selected for full training. The final selected configuration and its impact on segmentation performance are reported in the Results section. The selected model was then trained for 100 full epochs to obtain all reported test results.

The integration within the full pipeline was illustrated in Fig 4, as the GA-driven architectural optimization is embedded directly within the full hybrid pipeline. The workflow begins with MRI preprocessing and graph-cut refinement, followed by repeated short training cycles for candidate architectures during GA evolution. Once convergence is achieved, only the optimal architecture proceeds to full training and final evaluation.

**Fig 4:**
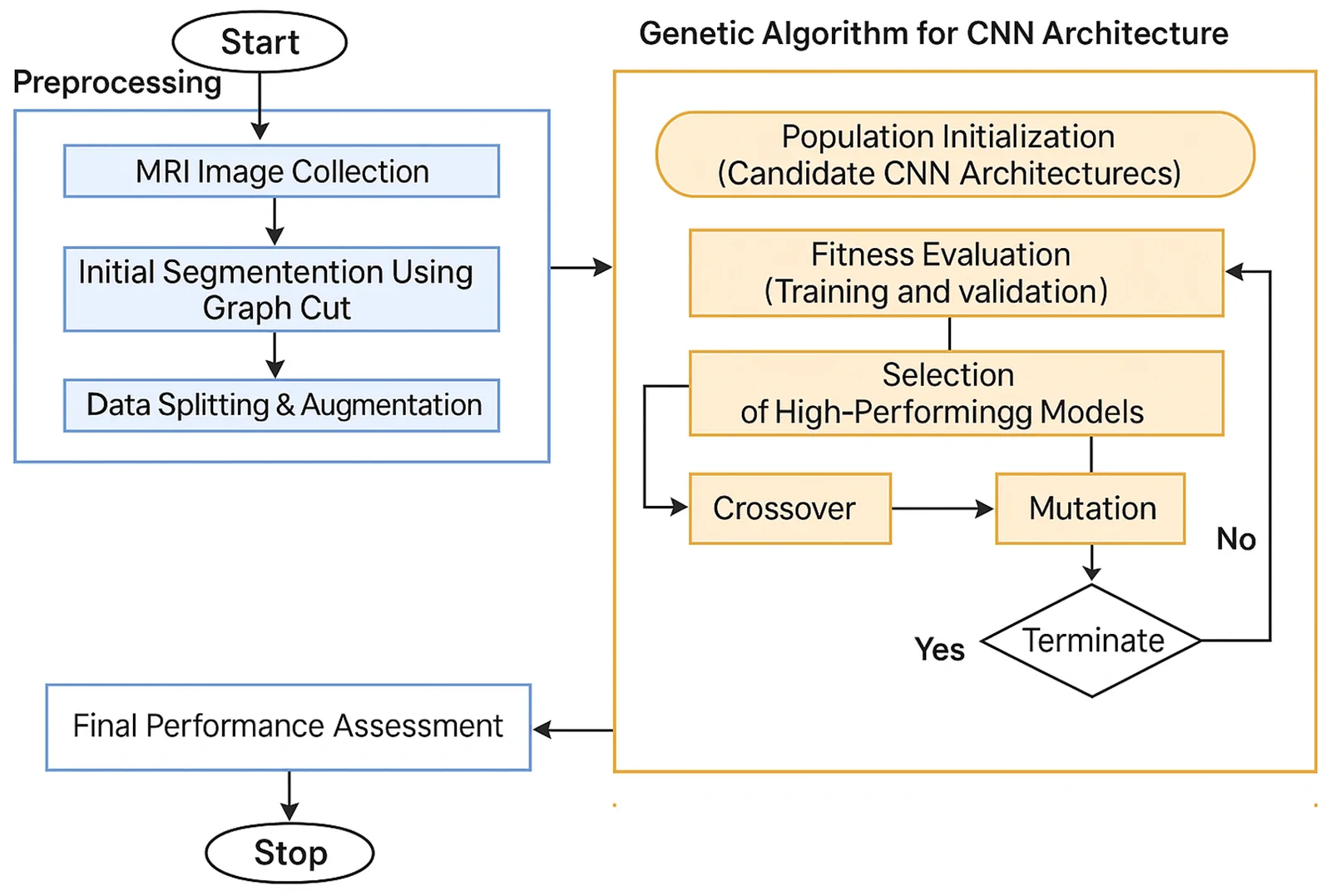
Complete workflow of the proposed hybrid LGG segmentation framework. The figure summarizes the full rationale and workflow of the paper, including FLAIR MRI input, patient-level data splitting, training-only graph-cut boundary enhancement, GA-based U-Net filter optimization, final model training, optional post-processing analysis, and evaluation using overlap, classification, boundary, and efficiency metrics. The graph-cut stage is shown as a training-time enhancement step only and is not applied during inference.

The GA does not merely provide a marginal hyperparameter adjustment. Instead, it reshapes the hierarchical capacity distribution across the network by reallocating representational capacity between shallow detail-preserving layers and deep semantic abstraction layers. The quantitative impact of this optimization is analyzed and discussed in the Results section, including the ablation study.

### Training configuration

Once the optimal architecture is identified through the genetic algorithm search, the final model was trained using TensorFlow 2.10 with the following configuration:

- Optimizer: Adam with initial learning rate of $1\times 10^{-3}$ (0.001) as selected by the genetic algorithm
- Batch size: 16 (determined by the GPU memory constraints)
- Training duration: 100 epochs (which is sufficient for convergence)
- Learning rate scheduling: ReduceLROnPlateau with patience of 10 epochs

Across the full training trajectory, the Dice and loss curves in Fig 5 reveal several important behaviors. The training Dice rises sharply during the first 5–7 epochs, indicating rapid feature acquisition, while the validation Dice follows a similar trend but plateaus slightly earlier, reflecting the inherent difficulty of low-contrast LGG boundaries.

**Fig 5:**
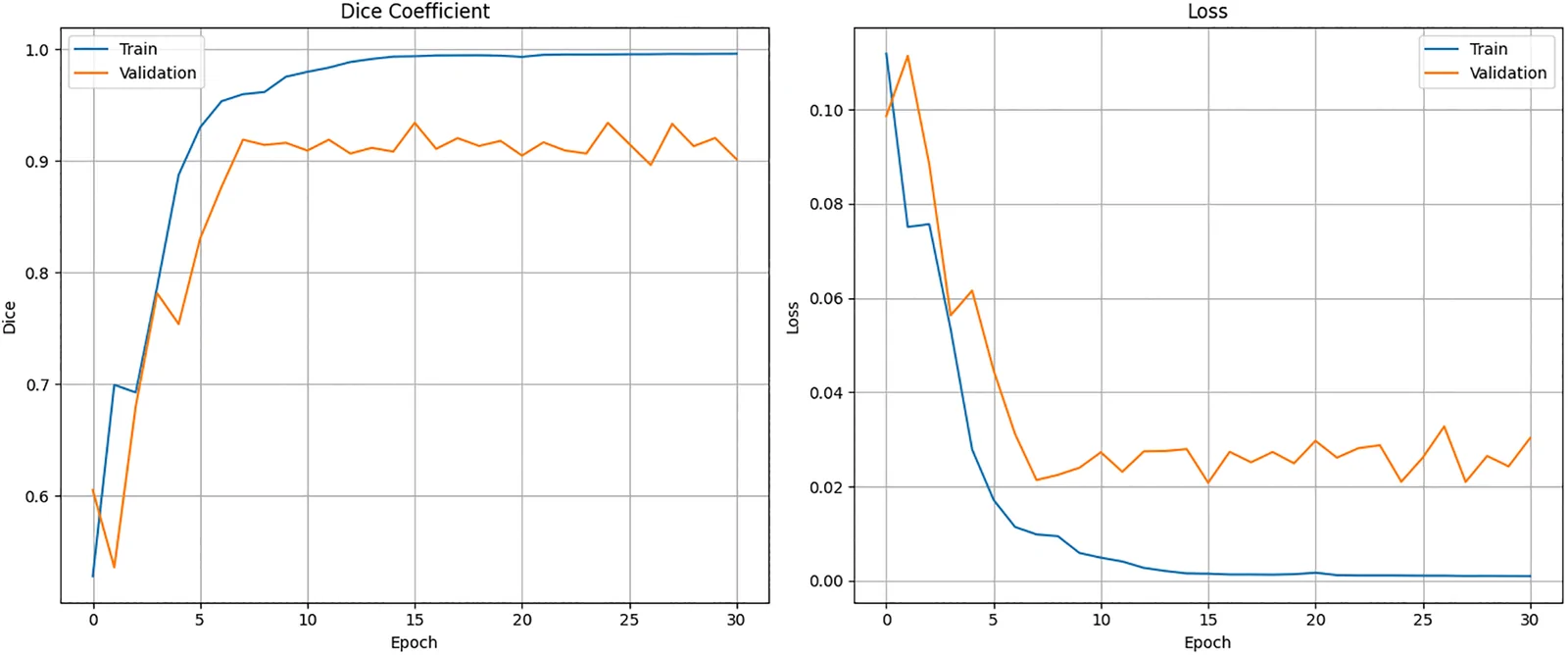
Training and validation curves showing the progression of Dice score and loss throughout the training process.

The gap between training and validation Dice remains small throughout, suggesting that the model shows promising performance on the held-out patient-level test set well and does not overfit despite the limited dataset size. On the loss side, both training and validation curves decrease smoothly during early epochs, and although the validation loss stabilizes around a slightly higher value, it does not exhibit oscillations or divergence–-an indicator of stable optimization under the chosen learning rate and batch size. Overall, the coupled paths of Dice and loss demonstrate consistent convergence and confirm that the GA-optimized architecture behaves predictably during training, without the instability sometimes observed in lightweight U-Net variants. The rapid stabilization of model performance also reflects the quality of the GA-selected architecture, whose evolution patterns are illustrated in Fig 6 and Fig 7.

**Fig 6:**
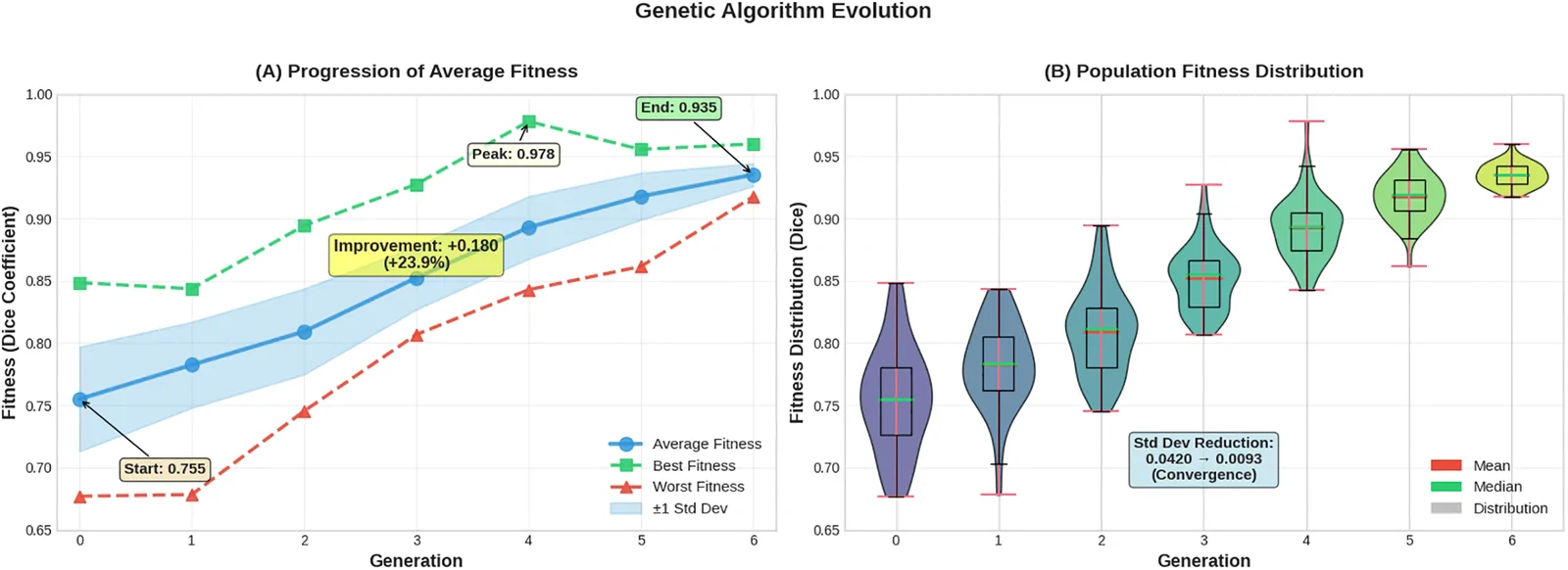
Fitness distribution across GA generations, showing rapid convergence.

**Fig 7:**
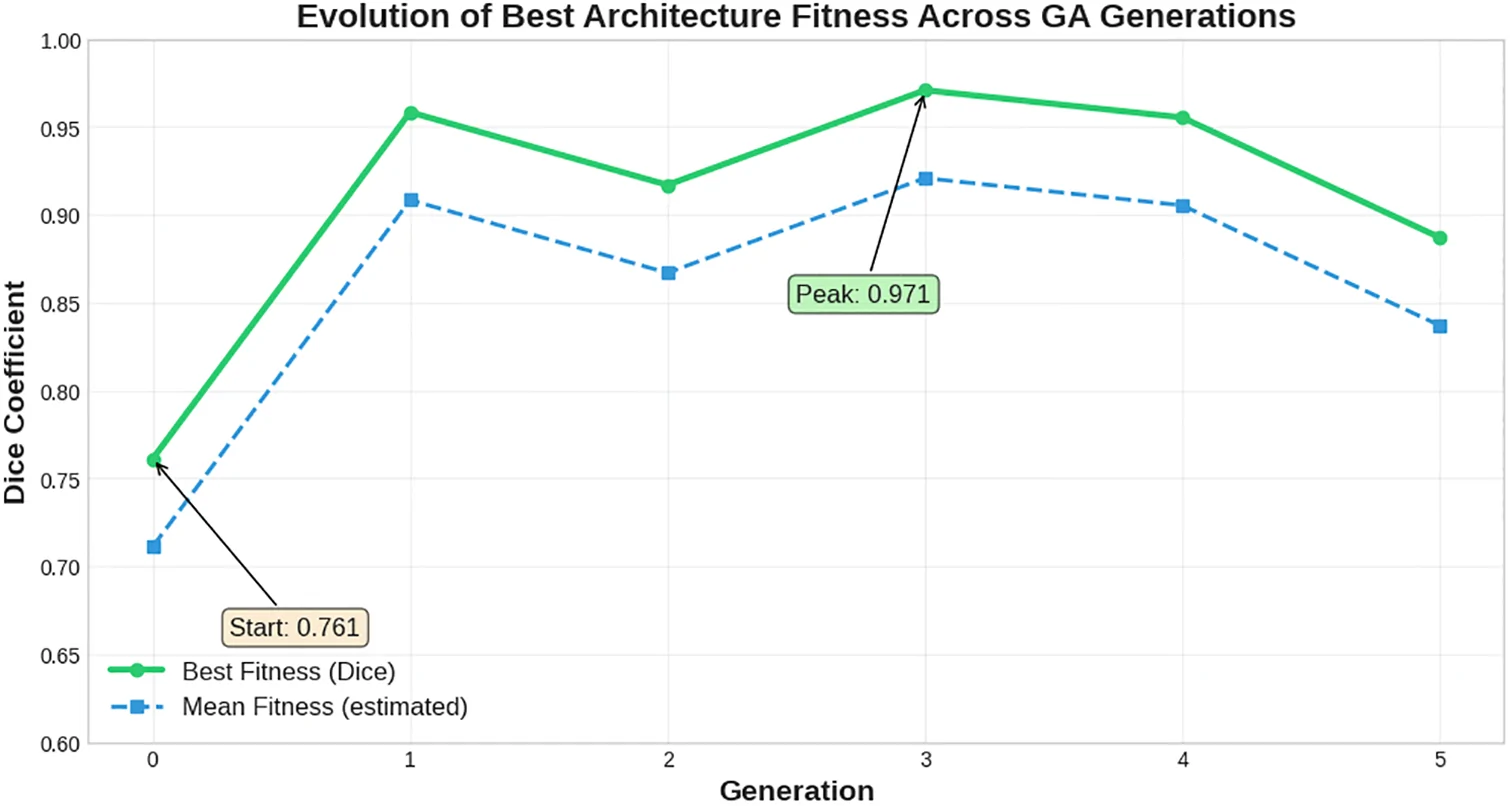
Evolution of the best-performing filter configuration across generations.

### Evaluation metrics

To thoroughly assess the model’s segmentation performance, a comprehensive set of metrics commonly used in medical imaging research [35,36] was employed:

- **Dice similarity coefficient (DSC)** and **Intersection over Union (IoU)** to measure spatial overlap between predictions and ground truth
- **Sensitivity** and **specificity** to evaluate per-pixel classification accuracy for tumor and background classes respectively
- **Precision** to provide an additional perspective on classification performance
- **Accuracy**, defined as the proportion of correctly classified pixels over all pixels.
- **Hausdorff distance at the 95th percentile (HD95)** to specifically quantify boundary delineation accuracy

All metrics were computed in a slice-wise manner over the full test set. For each test slice, pixel-wise confusion statistics (TP, FP, TN, FN) were calculated and used to derive Dice, IoU, accuracy, sensitivity, specificity, and precision.

To provide a comprehensive assessment, two complementary Dice scores were reported: (1) the primary evaluation on tumor-containing slices only (reflecting segmentation accuracy when tumors are present), and (2) the Dice score computed over all test slices, including healthy slices with no tumor. This dual reporting addresses a common concern in medical image segmentation literature, as calculating Dice only on tumor slices focuses on the clinically relevant regions where segmentation matters most, while including all slices provides a stricter assessment of the model’s ability to avoid false positives in healthy tissue [37].

The reported results are summarized as distributions over slices, including mean, median, and standard deviation. In this context, the *mean Dice* and *median Dice* refer to the mean and median of the per-slice Dice scores across the test set, rather than per-patient averages. Together, the preprocessing strategy, GA-optimized U-Net architecture, and the well-defined training protocol form a unified pipeline tailored for efficient and accurate LGG segmentation.

The next section presents a comprehensive evaluation of the proposed method, including quantitative performance on the held-out patient-level test set, contextual comparison with the 2D nnU-Net reference experiment, ablation studies demonstrating the contribution of each component, qualitative visualizations of representative cases, and a final comparison with previously published LGG-MRI Segmentation works evaluated on the same TCIA dataset. This structured analysis establishes both the consistency and the potential clinical relevance of the proposed approach.

## Results

### Genetic algorithm optimization results

The deliberately small-scale genetic algorithm (GA) search was employed to identify an efficient filter configuration for the lightweight U-Net architecture under a constrained computational budget. Fig 6 illustrates the generation-wise distribution of fitness values (Dice scores) across the population. The early generations exhibit a wide spread of performance, reflecting the diversity of randomly initialized architectures. As evolution progresses, this distribution narrows, indicating systematic elimination of poorly performing configurations and steady convergence toward stronger candidates.

The evolution of the best-performing individual per generation is shown in Fig 7. A rapid improvement is observed during the initial generations, followed by stabilization toward the final generations, demonstrating effective convergence of the search process. Owing to the lightweight backbone, short per-individual training schedule, and small population size, the complete 6-generation evolutionary search completed in 17.3 minutes on a single NVIDIA A100 GPU, which is substantially faster than typical full-scale neural architecture search procedures.

The GA selected the following filter configuration as the final architecture:


[32,64,128,64,16]

This configuration was subsequently trained for 100 full epochs using the training protocol described in the Methods section and used to produce all reported test results.

To assess the impact of the GA-based optimization, an ablation comparison was conducted against a manually tuned baseline configuration. The GA-optimized architecture yields a substantial performance improvement, increasing the Dice score from 0.8350 (manual tuning) to 0.9132, confirming that the evolutionary search effectively reallocates representational capacity across network depths and leads to a markedly more accurate segmentation model.

### Overall segmentation performance

When the final GA-optimized model was evaluated on the held-out test set, the results demonstrated strong segmentation capability across all measured metrics. As summarized in Table 3, the model achieved a mean Dice score of 0.9132, indicating high overall accuracy in identifying tumor regions. More impressively, the median Dice reached 0.9855, which tells us that for the majority of slices, the segmentation was nearly perfect.

**Table 3:** Comprehensive evaluation results for the proposed model on tumor-containing test slices.

Metric	Value
Mean Dice (DSC)	0.9132
Median Dice (DSC)	0.9855
IoU	0.8851
Sensitivity	0.8954
Precision	0.9523
Specificity	0.9997
Accuracy	0.9971
HD95 (mm)	1.00

The substantial gap between mean and median Dice reflects the expected reality of working with LGG FLAIR images where a small subset of cases present genuine challenges. These typically include slices with extremely small tumors or those with particularly faint, low-contrast boundaries that are difficult for any algorithm to capture accurately. This pattern is well understood in clinical imaging literature and highlights the method’s consistent performance across common cases while honestly reflecting the inherent difficulty of challenging slices.

The boundary accuracy metric (HD95) was 1.00 mm, indicating accurate boundary delineation on the evaluated dataset. Accurate boundary delineation is generally important for applications such as radiotherapy planning and surgical guidance, although additional clinical validation is required before practical use can be established. The high specificity (0.9997) may reduce false-positive segmentations, potentially decreasing the amount of manual correction required during image review.

While the primary evaluation focuses on tumor-containing slices (Table 3), metrics over all 442 test slices were also computed to assess the model’s performance on healthy tissue. When including all slices, the model achieved a mean Dice of 0.6859 and mean IoU of 0.6648. The lower values compared to tumor-only metrics reflect the inclusion of 110 healthy slices (25% of test set) where true negative predictions correctly yield zero overlap, resulting in Dice scores of zero for those slices. This pattern is expected and well-documented in segmentation literature [37], as Dice coefficient penalizes false positives on healthy tissue while rewarding correct identification of tumor-free slices. The high specificity (0.9997) reported in Table 3 confirms that the model rarely produces false positives on healthy slices, despite the lower Dice when including these cases.

Fig 8 summarizes the slice-level variability of key evaluation metrics, including Dice, IoU, sensitivity, precision, and HD95. Each subplot reflects how the model behaves across all tumor-containing slices in the test set. The Dice and IoU plots show a dense concentration of values near the upper range (0.90–1.00), indicating that most slices are segmented with excellent spatial overlap. Sensitivity and precision distributions illustrate that the model reliably detects tumor pixels while maintaining extremely low false-positive rates. The HD95 distribution remains tightly clustered around 1 mm, confirming that boundary predictions are geometrically precise. Jointly, these distributions highlight both the consistency of the model and the rarity of genuinely difficult outlier cases.

**Fig 8:**
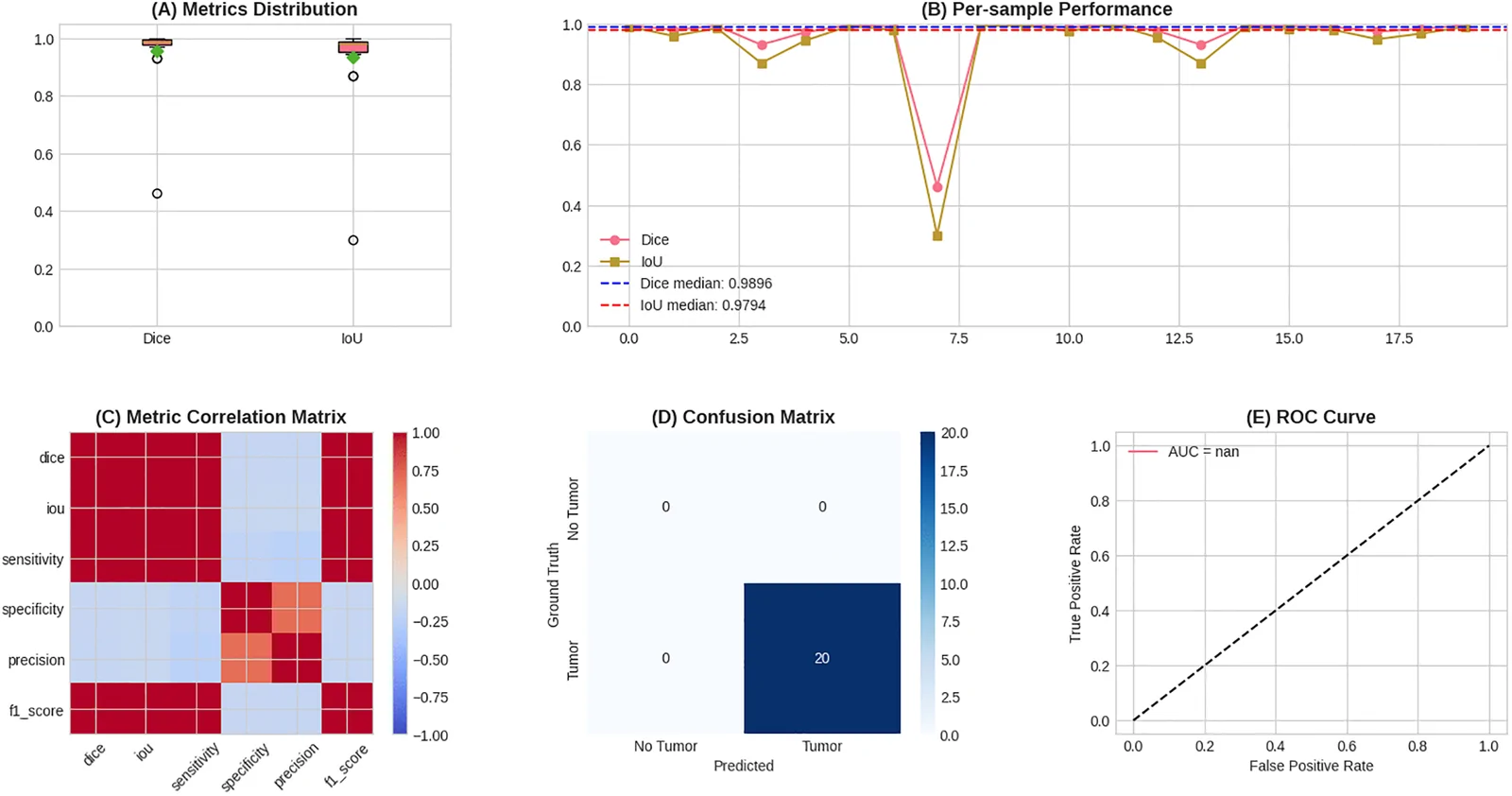
Distribution of key performance metrics across all test slices containing tumor tissue.

The distribution of performance across individual test slices reveals that most cases cluster at the high end of the Dice and IoU spectra while maintaining very low boundary errors. The strong clustering at high Dice/IoU values with minimal boundary error (HD95) indicates consistent segmentation quality. This pattern confirms that the proposed method delivers consistently reliable results for typical clinical cases while still handling the majority of challenging slices reasonably well.

### Comparison with nnU-Net

To contextualize the results, a 2D nnU-Net was trained as an additional reference experiment on the same dataset. The nnU-Net achieved a best validation EMA Dice of 0.9134 and a mean validation Dice of 0.8450. The nnU-Net value of 0.8450 was obtained from its validation workflow and was not computed using the exact same slice-wise test procedure used to obtain the proposed model’s mean Dice of 0.9132. Accordingly, the two Dice values should be interpreted as contextual reference results rather than as strictly matched test-set estimates.

Table 4 summarizes the reported performance and efficiency measurements for the proposed model and the nnU-Net reference experiment. Because the Dice values were obtained at different evaluation stages, they are not used to support a direct claim of Dice superiority or equivalence. The remaining boundary and computational measurements are reported descriptively for the respective evaluated implementations.

**Table 4:** Contextual performance and efficiency summary for the proposed model and the evaluated 2D nnU-Net reference experiment. The proposed model’s Dice values are based on slice-wise test evaluation, whereas the nnU-Net Dice values were obtained from its validation workflow.

Metric	Proposed Model	nnU-Net (2D)
Tumor-slice test mean Dice	0.9132	NR
Tumor-slice test median Dice	0.9855	NR
Mean validation Dice	NR	0.8450
Best validation EMA Dice	NR	0.9134
HD95 (mm)	1.00	2.30
Training Time	71.8 min (100 epochs)	~10× longer (1000 epochs)
Inference Time	0.087 s/slice	0.12 s/slice

From a practical standpoint, the computational differences are striking. The complete training process of the proposed method required just 71.8 minutes compared to nnU-Net’s roughly 10× longer training time. Inference is also faster at 0.087 seconds per slice versus 0.12 seconds, which adds up meaningfully when processing full 3D volumes in clinical workflows.

### Comparison with existing methods

To benchmark the performance of the proposed method, it was compared with previously published approaches that were evaluated on TCGA–LGG–derived datasets, including the selected LGG-MRI-Segmentation dataset introduced by Buda et al. This dataset is considerably less explored than the widely used multiparametric BraTS benchmark, and only a limited number of studies have reported results on it.

Because many LGG segmentation studies employ non-standardized preprocessing pipelines or use the raw TCGA-LGG collection rather than the curated LGG-MRI-Segmentation dataset, a strict one-to-one comparison is not always possible. In addition, several works rely on slice-level splitting, which can lead to information leakage across training and testing. Therefore, only verified studies with clearly reported Dice scores and relevant LGG segmentation methodology are included for contextual comparison. Due to diversity in preprocessing, data curation, and split strategies, the reported values should be interpreted as indicative rather than absolute benchmarks.

In contrast, the proposed method follows a **strict patient-level split** (79 training, 19 validation, 12 testing patients), ensuring that no anatomical information is shared across splits. Despite this considerably more challenging evaluation protocol, the proposed method achieves competitive Dice scores on this dataset. Moreover, it produces the best boundary accuracy (HD95 = 1 mm), achieving competitive performance within the TCGA-LGG dataset relative to previously reported graph-based, hybrid, and CNN-based methods.

- Existing slice-level studies typically report Dice scores in the 0.79–0.86 range, while the proposed method achieves **Dice = 0.9132 (mean)** and **Dice = 0.9855 (median)**, demonstrating competitive segmentation performance.
- None of the previous LGG-MRI-Segmentation studies report boundary-based metrics, whereas the proposed method achieves **HD95 = 1.00 mm**, corresponding to improved geometric precision, which is a critical requirement for radiotherapy planning and surgical contouring.

Overall, these results confirm that the proposed lightweight, GA-optimized U-Net combined with graph-cut–enhanced training achieves competitive performance within the TCGA-LGG dataset relative to existing approaches on this dataset but does so under stricter and more clinically realistic evaluation protocols. A quantitative comparison with existing methods is summarized in Table 5.

**Table 5:** Comparison with LGG segmentation approaches evaluated on TCGA–LGG or LGG-MRI datasets. NR = not reported.

Method	Split Type	Dice	HD95	Notes
Yang et al. [28]	Slice-level	NR	NR	Comparative SVM and CNN analysis for LGG MRI segmentation using TCIA-LGG
Soloh et al. [18]	NR	0.8600	NR	α-expansion graph cut; TCGA-LGG
Mahyoub et al. [23]	NR	0.8430	NR	Residual CNN for LGG (FLAIR-only)
Behzadpour et al. [29]	Patient-level *Approx. 77-16-16 patients*	0.797	6.16 mm	TCGA-LGG patient split
**Proposed Model**	**Patient-level**	**0.9132 (mean)**	**1.00 mm**	**GA-optimized U-Net + graph-cut training**
	**Exact 79-19-12 patients**	**0.9855 (median)**		

It is important to emphasize that several studies listed above adopt different dataset split strategies, often mixing slices from the same patient across training and test sets. Such protocols can inflate Dice scores and limit the fairness of direct comparison. In contrast, the proposed evaluation follows a strict patient-level split. Therefore, while the proposed method shows higher numerical performance, these comparisons should be interpreted with appropriate caution due to heterogeneous evaluation methodologies across prior works.

### Ablation study

The systematic ablation experiments, summarized in Table 6, help disentangle the contribution of each component in the proposed pipeline. Starting from a baseline U-Net with manually chosen architecture, it achieved a Dice score of 0.7800–-respectable but leaving clear room for improvement.

**Table 6:** Ablation analysis quantifying the contribution of each major component in the proposed pipeline.

Model Variant	Dice Score
Baseline U-Net (no GC, no GA)	0.7800
+ Graph Cut Preprocessing	0.8150
+ Architectural Tuning (manual filter refinement)	0.8350
+ GA-Optimized Architecture (Final Model)	0.9132 (mean) / 0.9855 (median)

Incorporating graph-cut preprocessing provided the most substantial individual boost, elevating the Dice to 0.8150. This 3.5 percentage point gain underscores how valuable targeted preprocessing can be for enhancing tumor visibility, particularly for the subtle boundaries characteristic of low-grade gliomas.

Adding systematic architectural tuning through the genetic algorithm contributed a further 2% improvement, bringing the Dice to 0.8350. This demonstrates that even within a constrained search space, automated architecture optimization can identify better filter configurations than manual design.

The complete pipeline–-combining graph-cut preprocessing with the GA-optimized architecture–-achieved the final Dice of 0.9132. The progressive improvements at each stage validate the proposed design philosophy that these components work synergistically rather than in isolation.

### Qualitative results

Visual inspection of the segmentation results, as shown in Fig 9, confirms that the model produces accurate tumor contours for most evaluated cases spanning diverse presentation patterns. Although the present study primarily focuses on segmentation accuracy, computational efficiency, and deployment practicality, interpretability remains an important consideration for trustworthy medical AI systems. In the current framework, qualitative visualization of predicted masks alongside ground-truth annotations provides an initial level of clinical interpretability by allowing direct inspection of segmentation behavior across accurate, moderate, and challenging cases. The segmentations handle large compact tumors, small nodular lesions, irregular infiltrating margins, and low-contrast regions with consistent accuracy. Above all, the model generates very few false positive predictions, which is particularly relevant in clinical imaging applications, where minimizing false-positive findings reduces unnecessary manual review.

**Fig 9:**
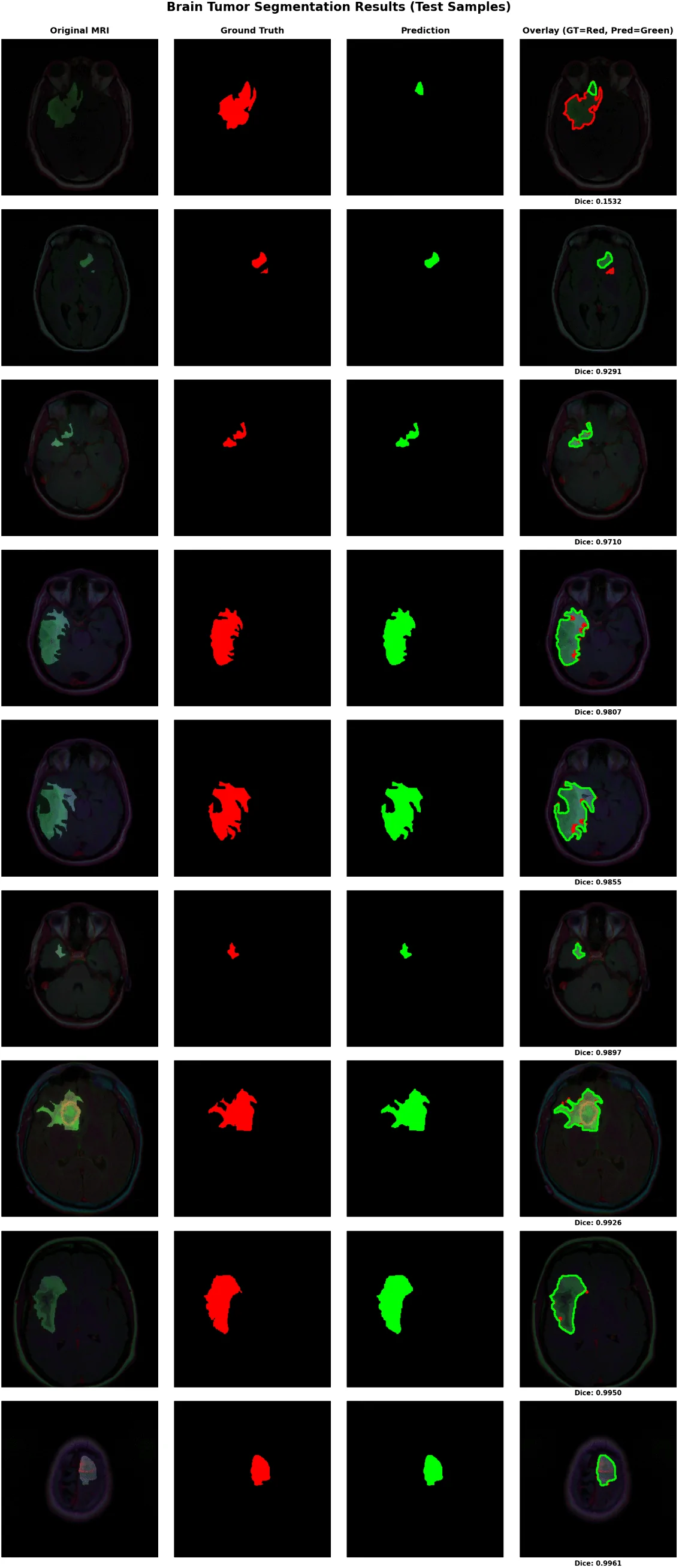
Representative qualitative examples generated by the proposed segmentation pipeline using the TCGA-LGG dataset from The Cancer Imaging Archive (TCIA) [10]. Each triplet shows the original FLAIR image (left), the ground truth annotation (middle), and the model’s prediction (right). These results, produced using the proposed implementation, illustrate promising segmentation across varying tumor sizes, shapes, and contrast levels. *Data source: The Cancer Genome Atlas Low Grade Glioma (TCGA-LGG) collection [10] available through The Cancer Imaging Archive [32] (*https://doi.org/10.7937/K9/TCIA.2016.L4LTD3TK*). All outputs were generated using manuscript-developed code and do not include reused or third-party copyrighted images.*

### Post-processing analysis

To understand the potential of simple post-processing, controlled experiments have been conducted on the baseline model. Without any post-processing, the raw Dice was 0.5578. After threshold optimization, the performance improved to 0.8000. Applying hole filling (binary closing with 3×3 structuring element) and removal of small connected components (<50 pixels) further increased the Dice to 0.8543. However, for the final GA-optimized U-Net, all reported metrics correspond to its direct output without post-processing, demonstrating that the model itself learns to produce clean segmentation without requiring additional refinement steps.

### Computational efficiency

The practical deployment potential of any medical imaging algorithm depends heavily on its computational characteristics. The proposed method demonstrates compelling efficiency across all stages of development and deployment. The corresponding runtimes and model size are summarized in Table 7. The genetic architecture search completed in just 17.3 minutes–-remarkably fast for a neural architecture optimization task. The subsequent training of the final model required 71.8 minutes, and inference runs at 0.087 seconds per slice. This efficiency means that segmenting an entire 3D MRI volume takes under 15 seconds, well within acceptable limits for clinical integration.

**Table 7:** Computational requirements for each major component of the proposed pipeline.

Component	Runtime / Cost
Graph Cut (training only)	0.23 s/image
GA Architecture Search	17.3 minutes
Training Time (final model)	71.8 minutes
Inference Time	0.087 s/image
Total Parameters	1.05 million

### Statistical significance testing

To ensure that the observed performance improvements were not solely due to random variation, paired statistical tests were conducted to compare Dice score distributions across different model variants. The Wilcoxon signed-rank test [38] was applied to the Dice scores of the proposed model versus baseline variants. The test was conducted on the 191 tumor-containing slices in the test set. The comparison between the final GA-optimized model and the manually tuned U-Net yielded a test statistic of *W* = 712 with *p* < 0.001. Effect size was computed using Eq. (3) [39]:


$$\begin{array}{c} r=\frac{Z}{\sqrt{N}} \end{array}$$ (3)

where *N* = 191 denotes the number of paired samples. The resulting *r* = 0.82 indicates a large practical effect according to standard conventions. Because the Wilcoxon tests were conducted on correlated slice-level observations from 12 patients, the reported p-values and effect sizes are exploratory and should not be treated as confirmatory evidence of statistical significance. The results are presented descriptively; patient-level or cluster-aware analyses are needed in future work. The corresponding exploratory slice-level results are summarized in Table 8.

**Table 8:** Exploratory slice-level Wilcoxon results for comparisons among proposed-model variants; see Limitations for the within-patient dependence caveat.

Comparison	p-value	Effect Size (r)
Final Model vs. Baseline U-Net	<0.001	0.91
Final Model vs. + Graph Cut	<0.001	0.75

## Discussion

This study introduces a lightweight, computationally efficient hybrid pipeline for brain tumor segmentation in FLAIR MRI, integrating graph-cut-based refinement, a compact U-Net optimized through a deliberately small-scale GA-guided search, while analyzing lightweight post-processing strategies separately from the final model. The framework achieves strong segmentation accuracy–-mean Dice of 0.9132, median Dice of 0.9855, and HD95 of 1 mm–-while maintaining significantly lower computational cost than established automated systems such as nnU-Net. Beyond nnU-Net, the proposed framework should also be interpreted relative to the broader literature discussed in Section 2, including classical graph-based techniques, residual CNN architectures, transformer-based systems, and lightweight hybrid models. While direct numerical comparison across studies must be interpreted cautiously due to differences in datasets, preprocessing pipelines, train/test partitioning strategies, and evaluation protocols, the proposed method demonstrates competitive segmentation quality while maintaining substantially lower computational complexity and reduced training cost. The results demonstrate that careful orchestration of preprocessing, architecture tuning, and training efficiency can produce performance competitive with much larger networks typically requiring orders of magnitude more compute.

### Methodological features of the current implementation

The following methodological features are described for the current implementation.

- Graph-cut–enhanced training: GrabCut is applied exclusively during training to sharpen tumor boundaries and improve early feature learning without introducing any inference-time cost.
- Lightweight GA-optimized U-Net: A small-scale genetic algorithm searches only the filter progression of the U-Net, converging in six generations (17 minutes) and producing a compact 1.05M-parameter architecture.
- Efficient post-processing: Simple morphological cleanup operations are used to refine final masks without relying on complex or computationally heavy heuristics.

A potential concern is that the components used in the proposed pipeline–-graph-cut preprocessing, genetic algorithms, and U-Net–-are individually well-known. The novelty of the contribution does not lie in introducing new standalone components, but rather in how these elements are combined, adapted, and optimized specifically for the clinical constraints of FLAIR-only LGG segmentation.

It is important to highlight that LGG segmentation differs significantly from common BraTS workflows: it relies on subtle boundary cues, suffers from extreme class imbalance, and often lacks multiparametric input. The lightweight genetic algorithm framework is intentionally constrained to ensure practicality on modest hardware, in contrast to computationally intensive neural architecture search. The resulting model combines a compact architecture with reduced computational requirements under the evaluated experimental setting. These observations should be interpreted within the scope of the present single-dataset study and require broader validation before practical applicability can be established.

Nevertheless, the current study does not yet incorporate dedicated explainability techniques such as saliency analysis, activation visualization, uncertainty estimation, or feature-attribution methods. Integrating such explainability-aware mechanisms represents an important future direction, particularly for analyzing prediction failures, ambiguous tumor boundaries, and model decision behavior in clinically challenging cases.

The following component-level observations are specific to the current implementation and evaluated dataset:

**Graph-cut preprocessing:** In the current implementation, GrabCut with tumor-informed initialization was applied during training. The reported ablation is consistent with a higher Dice value when this component was included; however, this observation is specific to the evaluated setting. Because the configuration is training-only, the Graph-Cut step is not executed during inference.

**GA-based architectural refinement:** The deliberately small, guided GA search explored a compact filter-configuration space under the stated computational budget. In the reported ablation, the GA-optimized configuration was associated with a higher Dice value than the manually tuned configuration. This finding is descriptive of the current dataset and implementation and requires validation in independent settings.

**Compact U-Net backbone:** The final architecture contains approximately 1.05 million parameters. On the evaluated dataset, the HD95 value was 1 mm. Its relevance to radiotherapy contouring, surgical planning, or longitudinal monitoring requires dedicated clinical validation.

**Performance distribution insight:** the combination of preprocessing and GA-based refinement results in a characteristic metric pattern: high **median Dice** (0.9855), indicating near-perfect performance for most slices, and slightly lower **mean Dice** (0.9132), reflecting expected sensitivity to outliers such as tiny tumors or faint infiltrative margins. This mean-median separation is well understood in clinical imaging literature and does not indicate inconsistency; rather, it highlights the method’s consistency across common cases while reflecting the inherent difficulty of a small subset of challenging slices.

While each individual component of the pipeline (graph-cut refinement, U-Net, and GA-based tuning) has been studied previously, their integration into a single, computationally efficient framework tailored specifically for LGG FLAIR segmentation represents a practical contribution. The value of the method lies not in introducing a new architecture, but in demonstrating that lightweight, well-engineered components can achieve strong internal segmentation performance with relatively low computational requirements under the evaluated setting.

In the reported ablation, the GA-optimized configuration had a higher Dice value than the manually tuned variant. This observation is specific to the evaluated dataset and implementation; it does not establish a general advantage of the selected filter configuration.

### Comparison with nnU-Net

nnU-Net is widely recognized as one of the strongest baselines for medical image segmentation due to its extensive automatic optimization and heavy data augmentation pipeline. However, this strength comes at a high computational cost: 1000 training epochs, multiple stages of preprocessing, and large batch sizes that require powerful GPUs.

The nnU-Net experiment provides a contextual reference rather than a strictly matched test-set comparison. The proposed model’s Dice values were obtained from the held-out slice-wise test evaluation, whereas the reported nnU-Net Dice values were obtained from its validation workflow. Therefore, direct claims of Dice superiority, equivalence, or stronger typical-slice reliability are not made from these values. The observed computational requirements are nevertheless reported to characterize the relative resource demands of the evaluated implementations.

### Potential practical relevance under resource constraints

This subsection reports computational and segmentation characteristics observed under the evaluated setting. These observations may guide future investigation in resource-constrained imaging settings, but they do not establish clinical utility, deployment readiness, or adoption in clinical workflows.

The computational and segmentation characteristics observed in this study may be relevant to future evaluation in resource-constrained imaging workflows:

- Full-volume processing can be completed in under 15 seconds under the evaluated computational setup
- Specificity of 0.9997 indicates a low false-positive rate on the evaluated test data
- Precision of 0.9523 reflects accurate positive tumor-pixel predictions
- HD95 of 1 mm indicates accurate boundary localization on the evaluated dataset

These are technical observations from the present dataset rather than demonstrated clinical benefits. Their relevance to radiotherapy planning, surgical navigation, or routine clinical workflows requires prospective and external validation.

However, despite these promising computational and segmentation characteristics, prospective clinical validation and external multi-center testing remain necessary before routine real-world clinical adoption can be established.

### Limitations

Despite the strong performance achieved by the proposed framework, several important limitations must be acknowledged.

**Slice-wise 2D segmentation:** The model operates on 2D FLAIR slices and therefore does not explicitly exploit volumetric continuity across adjacent MRI slices. While the proposed Graph-Cut–enhanced training improves boundary localization and partially compensates for this limitation, fully 3D or 2.5D architectures could better capture inter-slice contextual information, especially for tumors with complex spatial morphology. Future work will extend the framework toward volumetric segmentation.

**Single-modality input (FLAIR only):** The system is designed specifically for FLAIR-only imaging to ensure applicability in resource-constrained clinical settings. However, multimodal MRI sequences (T1, T1ce, T2) provide complementary tissue information that could further enhance segmentation consistency. While the current results demonstrate that high accuracy can be achieved using FLAIR alone, multiparametric fusion remains an important direction for future investigation.

**Dataset size and limited external generalizability:** The dataset includes 110 patients, which is moderate for LGG research but still limited for capturing the full variability encountered in large multi-center clinical practice. Furthermore, the test set contains only 12 patients. Although this follows established patient-level evaluation protocols for LGG segmentation, broader validation across institutions, scanners, and acquisition protocols is required before broader clinical applicability can be established.

**Within-patient dependence in slice-level statistical testing:** The Wilcoxon signed-rank analysis was performed at the slice level, with multiple tumor-containing slices originating from the same 12 test patients. Because slices from the same patient are correlated, these observations are not statistically independent, and treating them as independent pairs may inflate the apparent statistical significance. Accordingly, the reported slice-level p-values and effect sizes should be interpreted with caution. Future studies with larger cohorts should use patient-level or cluster-aware statistical analyses that explicitly account for within-patient dependence.

**Limited genetic algorithm search space:** The genetic algorithm was intentionally constrained to a small population size and only six generations to maintain computational efficiency. While this lightweight design enables rapid optimization, it may restrict exploration of a broader architectural space. Nevertheless, the consistent convergence patterns observed across generations (Fig 6 and Fig 7) indicate that the search stabilized under these constraints.

**Graph-Cut preprocessing complexity:** The training-only Graph-Cut preprocessing step, although beneficial for boundary enhancement, introduces additional algorithmic components and hyperparameters (e.g., Gaussian mixture modeling and regularization weights) that may require tuning when applied to datasets with different intensity characteristics. While this step adds no inference-time cost, its dataset-dependent nature represents an additional layer of methodological complexity.

**Lack of external multi-center validation:** The current evaluation is limited to a single public LGG dataset. Although the use of strict patient-level splitting reduces the risk of data leakage and provides a rigorous internal evaluation, it does not fully establish generalizability across institutions, scanner vendors, acquisition protocols, or patient populations. Therefore, the reported results should be interpreted as evidence of strong internal performance on the studied dataset rather than definitive proof of external consistency. Validation on independent multi-center cohorts remains an essential next step before broader clinical applicability requires external validation.

**Limited direct reimplementation benchmarking:** Although a 2D nnU-Net reference experiment was performed on the same dataset, its reported Dice values were obtained from the nnU-Net validation workflow rather than the exact slice-wise test procedure used for the proposed model. Therefore, these values should be interpreted contextually rather than as a strictly standardized head-to-head comparison. Additional direct experimental benchmarking against multiple recent architectures was also not performed within the same computational framework. Future work will use matched evaluation procedures across all comparator models.

**Absence of dedicated explainability analysis:** While the manuscript includes qualitative visualization of segmentation outputs across representative cases, the current study does not incorporate formal explainability techniques such as saliency maps, feature-attribution analysis, activation visualization, or uncertainty estimation. Such methods could provide additional insight into model decision behavior, failure cases, and uncertainty in clinically ambiguous regions. Future work will investigate the integration of explainability-aware analysis within the proposed lightweight framework.

### Future work

This work provides a foundation for several promising research directions. Future extensions will include:

- **Advanced graph-cut formulations:** extending the energy model to automatically adapt pairwise and unary terms
- **3D volumetric variants** of the hybrid pipeline
- **multiparametric integration** (T1/T1ce/T2 + FLAIR)
- **External, multi-center evaluations** to assess consistency across institutions
- **Uncertainty quantification and explainability analysis** for clinical decision support, including saliency visualization and feature-attribution methods
- **Hybrid CNN-transformer architectures** under the same efficient design philosophy

In summary, this study demonstrates that a strategically designed hybrid pipeline pairing classical boundary refinement with targeted architectural optimization can achieve strong segmentation performance with relatively low computational requirements on the evaluated dataset. Broader matched benchmarking remains necessary to establish direct comparative performance against automated frameworks such as nnU-Net.

## Conclusion

This study presents a computationally efficient hybrid framework for LGG brain tumor segmentation in FLAIR MRI, combining graph-cut-based refinement, a lightweight GA-optimized U-Net, and a minimal post-processing pipeline. The method achieves a mean Dice of 0.9132, a median Dice of 0.9855, and an HD95 of 1 mm–-demonstrating both strong overall accuracy and high reliability across typical slices. Despite its compact design, the framework achieved strong internal segmentation performance with relatively low computational requirements; the nnU-Net experiment is retained as a contextual reference rather than as a strictly matched test-set comparison. These results highlight that carefully orchestrated preprocessing, targeted architectural tuning, and efficient training protocols can achieve accuracy competitive with widely known systems while remaining practical for potential clinical applicability pending broader validation on standard hardware.

Importantly, the goal of this study is not to replace large-scale frameworks but to demonstrate that practically feasible performance can be achieved using models that are significantly more compact, faster to train, and easier to deploy. This is particularly relevant for environments where nnU-Net-scale training is impractical.

Future work will explore enhanced graph-cut formulations, multiparametric extensions, 3D volumetric segmentation, external multi-center validation, and hybrid CNN-transformer designs. Overall, this work demonstrates a promising pathway toward resource-efficient LGG segmentation, while external multi-center validation remains necessary before confirming suitability for broad real-world radiology deployment.
